# Mechanistic insights into TNFR1/MADD death domains in Alzheimer’s disease through conformational molecular dynamic analysis

**DOI:** 10.1038/s41598-021-91606-4

**Published:** 2021-06-10

**Authors:** Mubashir Hassan, Sara Zahid, Hany Alashwal, Andrzej Kloczkowski, Ahmed A. Moustafa

**Affiliations:** 1 Institute of Molecular Biology and Biotechnology, The University of Lahore, Lahore, Pakistan; 2 College of Information Technology, United Arab, Emirates University, Al-Ain, 15551 UAE; 3 Battelle Center for Mathematical Medicine, Nationwide Children’s Hospital, Columbus, OH 43205 USA; 4 Department of Pediatrics, The Ohio State University College of Medicine, Columbus, OH 43205 USA; 5 Department of Human Anatomy and Physiology, the Faculty of Health Sciences, University of Johannesburg, Johannesburg, South Africa; 6 MARCS Institute for Brain and Behaviour, Western Sydney University, Sydney, NSW Australia; 7 School of Psychology, Western Sydney University, Sydney, NSW Australia

**Keywords:** Biochemistry, Biophysics, Computational biology and bioinformatics, Structural biology

## Abstract

Proteins are tiny players involved in the activation and deactivation of multiple signaling cascades through interactions in cells. The TNFR1 and MADD interact with each other and mediate downstream protein signaling pathways which cause neuronal cell death and Alzheimer’s disease. In the current study, a molecular docking approach was employed to explore the interactive behavior of TNFR1 and MADD proteins and their role in the activation of downstream signaling pathways. The computational sequential and structural conformational results revealed that Asp400, Arg58, Arg59 were common residues of TNFR1 and MADD which are involved in the activation of downstream signaling pathways. Aspartic acid in negatively charged residues is involved in the biosynthesis of protein. However, arginine is a positively charged residue with the potential to interact with oppositely charged amino acids. Furthermore, our molecular dynamic simulation results also ensured the stability of the backbone of TNFR1 and MADD death domains (DDs) in binding interactions. This DDs interaction mediates some conformational changes in TNFR1 which leads to the activation of mediators proteins in the cellular signaling pathways. Taken together, a better understanding of TNFR1 and MADD receptors and their activated signaling cascade may help treat Alzheimer’s disease. The death domains of TNFR1 and MADD could be used as a novel pharmacological target for the treatment of Alzheimer’s disease by inhibiting the MAPK pathway.

## Introduction

Alzheimer’s disease (AD), a communicable neurodegenerative form of dementia, is associated with a deposition of beta amyloid (Aβ), destruction of neuronal synapsis, neurofibrillary tangle (NFT), and neuronal apoptotic cell death^1–4^. Specifically, neurofibrillary tangles, neuropil threads, tau protein hyperphosphorylation, glial activation and amyloid plaques are regarded as positive hallmark characteristics whereas negative lesions promote neuronal and synaptic damage in AD^5^.

Among all of these pathological features, neuroinflammation has been the foremost contributor to AD which is best demonstrated via TNF mediator^5^. The tumor necrosis factor-alpha (TNF-α) is a pleiotropic inflammatory cytokine known as a member of the TNF superfamily of ligands orchestrated with a master regulator of innate and adaptive immune system in several neurodegenerative impairments^6^. The TNF cellular response is mediated via two perceptive receptors: the TNF receptor 1 (TNFR1) with a molecular weight of of 55 kDa and TNF receptor 2 (TNFR2) with a molecular weight of of 75 kDa^7,8^. Predominantly, TNFR1 upregulates inflammatory responses and TNFR2 facilitates neuroprotection, regeneration as well as homeostasis. The determination of TNFR1 expression in microglial cells of post-mortem brain tissue of AD patients is the first possible indication of AD neuroinflammation. This elevated TNF expression feature in the brains of AD patients is due to resident microglial failure to efficiently phagocytose Aβ^9^.

Multiple signaling cascades crosstalk with apoptotic neuronal cell death in AD progression. The implication of TNF-α and mitogen-activated protein kinase/c-Jun N terminal kinase (MAPK/JNK) signaling pathways systematically describes the neuronal cell death in chronic AD^10^. This mechanism involves the TNF receptor superfamily member 1A (TNFRSF1A) which induces microglial activation and Aβ fibril formation in the brain. The TNFR-associated death domain (TRADD) binds to MADD (MAPK activating death domain) which ultimately interacts with TNFR1^11^. The eventual crucial binding of TNFR1 causes the activation of Fas-associated death domain (FADD), receptor-interacting protein (RIP) and TNFR-associated factor (TRAF2) resulting in microglial cell apoptosis^12^. The transcription factors JNK and NF-kB are upregulated recruiting IKK complex and production of Aβ1-42 via caspase-3 and caspase-8 induce neuronal apoptosis in the brains of AD patients. However, the downregulated expression of TNFR1 protein is observed in AD patients which involves FADD like IL-1β converting enzyme (FLICE) inhibitor protein (FLIP) and TNFR2. Therefore, this work aims to explore the interactive residues of both signaling proteins and mediated signaling pathways.

In the current study, molecular docking and conformational dynamic simulation approaches were employed to explore the active binding site residues of both TNFR1 and MADD and their involvement in the development of AD through the activation of signaling pathways. The death domains (DDs) of both MADD and TNFR1 were accessed through homology modeling and protein data bank, respectively. Sequence and structural analyses against selected DDs were performed using various computational tools and online servers, respectively. The interactive residues in DDs binding were observed through protein–protein docking and stability of generated docked complexes were confirmed through MD simulation. A better understanding of DDs signaling cascade may help cure AD using TNFR1/MADD as novel targets.

## Computational methodology

### Retrieval of human death domains (DDs) sequences

The amino acids sequences of the death domain of MAP kinase-activating death domain (MADD: Q8WXG6) protein with residue range 1340–1415 (76 AA) and TNF Receptor 1 (TNFR1A; P19438) with (AA 356–441; 86) were accessed from the Uniprot Knowledgebase database (https://www.uniprot.org/). The conservation pattern of both TNFR1 and MADD death domains were observed in different organisms by BLAST (https://blast.ncbi.nlm.nih.gov/Blast.cgi). Multiple sequence alignment (MSA) of human TNFR1 and MADD domains was performed using Clustal Omega from EMBL-EBI Web Services^13^ and conserved residues in all selected organisms were visualized using the AliView software^14^.

### Repossession and structure prediction of DDs

The death domains of TNFR1 and MADD structures are required to observe the interactive behavior of both death domains. The death domain of TNFR1 was retrieved from the Protein Data Bank (www.rcsb.org/1ich) by PDB accession code: 1ICH. However, the interactive partner MADD the crystal structure of human MADD (death domain) is not available in the PDB. Therefore, a homology modeling-based approach was employed to predict the MADD protein domains separately. Four different domains (uDENN, cDENN, dDENN and Death Domain) were predicted using the Swiss modeling server^15^. The constructed and accessed target protein death domain structures were further minimized by employing a conjugate gradient algorithm and AMBER force field using the UCSF Chimera 1.10.1^16^. Furthermore, the MolProbity server^17^ and ProSA-web^18^ were utilized to assess the stereochemical properties of targeted structures. The ProtParam tool was employed to predict theoretically (isoelectric point) *PIs*, extinction coefficients, aliphatic and instability indexes, and GRAVY values for proteins^19^. Moreover, Ramachandran plots and values were obtained from MolProbity server^20^. The Discovery Studio 4.1 Client^21^, which is a visualizing tool, was used to generate the hydrophobicity graph. The overall protein architecture and statistical percentage values of helices, beta-sheets, coils and turns were retrieved from the online server VADAR 1.8^22^.

### Molecular docking assay

Protein–protein docking is a significant computational approach used to explore the interacting residues and their functional involvement in downstream signaling pathways^23,24^. The conformational and interactive behavior of death domains was evaluated through protein–protein docking servers. Initially, docking binding pocket residues were identified by using an online server DEPTH to predict the residue depth^25,26^ This server depicted the propensity of particular amino acids being involved in the formation of the binding pocket. After that, the following three different online protein–protein docking servers were employed to predict the core residues that may be involved in the cellular functions and biological processes in downstream signaling pathways: ZDOCK^27^, ClusPro^28^ and HawkDock^29^.

The ZDOCK version 3.0.2 was used to generate rigid-body docking conformations with the default search parameters. ZDOCK uses Fast Fourier Transform (FFT) correlation-based method and predicts favorite conformational positions in docked complexes. In ZDOCK, conformations are predicted based on desolvation and electrostatic contributions along with pairwise shape complementarity. The protein–protein docking analysis of targeted protein structures was carried out by ClusPro 2.0 server to acquire the best native conformation. ClusPro 2.0 generates4 categories of predicted models: (1) Balanced, (2) Electrostatic-favored, (3) Hydrophobic-favored and (4)van der Waals = electrostatics. All categories and models were ranked by cluster size and the best models were selected from all categories. We discarded the complexes with unacceptable penetrations of receptor atoms to the atoms of the ligands. Finally, the remaining candidates were ranked according to the geometric shape and complementarity scores.

Another docking experiment was performed using the HawkDock server. Both proteins were uploaded and default server parameters were used. It has been observed that a lower value of energy corresponds to a better HawkDock score^29^. The HawkDock server generates several models of a docked complex and ranks them by assigning HawkDock scores in ascending order. For each of the docked complexes along with respective targets, the model 1 (top model) was taken for further analysis based on scoring value. The bonding interaction pattern between DDs for all models generated by docking servers was analyzed by using Discovery Studio (4.1) and UCSF Chimera 1.10.1, respectively.

### Molecular dynamics (MD) simulations

To understand the residual backbone flexibility of protein structure, MD simulations were carried out by Groningen Machine for Chemicals Simulations package (GROMACS 4.5.4)^30^, with the GROMOS 96 force field^31^. The overall system charge was neutralized by adding ions. The steepest descent approach (1000 ps) for each protein structure was applied for energy minimization. For energy minimization, the nsteps = 50,000 were adjusted with energy step size value (emstep) 0.01. Particle Mesh Ewald (PME) method was employed for energy calculation ofelectrostatic and van der Waals interactions; the cut-off distance for the short-range vdW potential (rvdw) was set to 14 Å, whereas neighbor list (rlist) and nstlist values were set to 1.0 and 10, respectively, in em.mdp file^32^. This permits the use of the Ewald summation at a computational cost comparable with that of a simple truncation method of 10 Å or less, and the linear constraint solver (LINCS)^33^ algorithm was used for covalent bond constraints with the time step set to 0.002 ps. Finally, the molecular dynamics simulation was carried out for 50 ns with 25,000,000 nsteps in md.mdp file. Different structural evaluations such as root-mean-square-deviations and fluctuations (RMSD/RMSF), solvent accessible surface areas (SASA) and radii of gyration (Rg) of back bone residues were analyzed using the 2D plotting tool Xmgrace (http://plasma-gate.weizmann.ac.il/Grace/) and UCSF Chimera 1.10.1 program for the interactive visualization and analysis of molecular structures.

## Results

The generated computational results showed the conservation behavior of both DDs sequences in different species. This conservation pattern depicted the core residues of DDs which may have a functional role in the binding interaction of TNFR1 and MADD. In DDs docking results, three common residues (Asp400, Arg58, Arg59) were observed and may work as a connector between DDs. Furthermore, the stability of docked complexes has been verified through RMSD/F, Rg and SASA graphs. The proposed mechanistic pathways ensure the significance of TNFR1 and MADD interactions that could be used as a novel target for development of drugs for the treatment of Alzheimer’s disease.

### Residue conservation analysis of DDs

**Figure 1:**
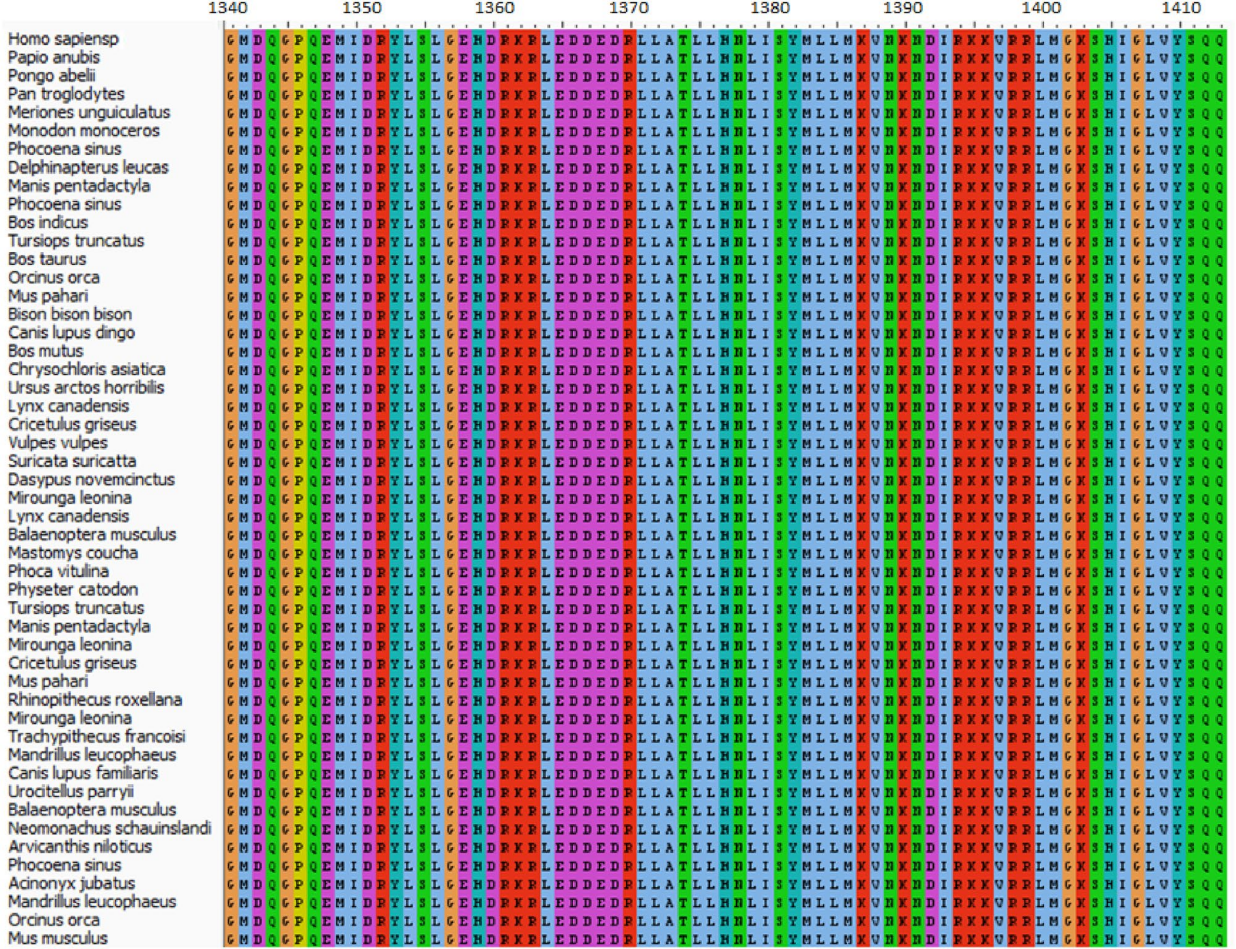
Residue conservation pattern of MADD.

**Figure 2:**
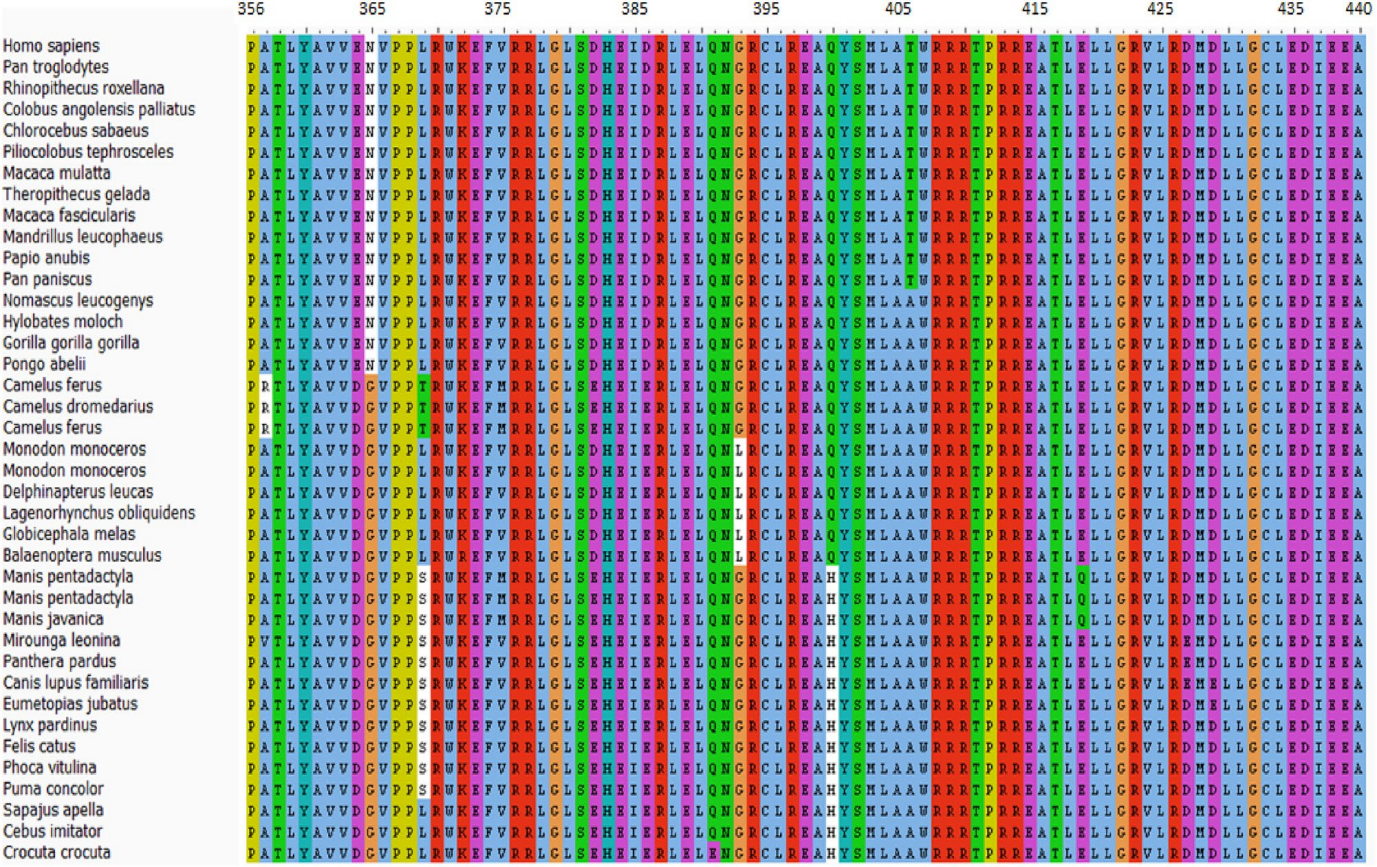
Residue conservation pattern of TNFR1 death domain in selected organisms.

The retrieved amino acid sequences of both death domains were aligned in multiple species to check the conversation pattern. The MSA generated results depicted that both MADD and TNFR1 death domains were conserved in all selected organisms. This residue conservation pattern may indicate their functional involvement in downstream signaling pathways (Figs. 1 and 2). In the MADD protein, the death domain is located between residues 1340 and 1410. The amino acids in this domain are the most important for the interaction with the death domain of TNFR1 are located between residues 356 and 440. In TNFR1, the death domain residues around position 357 in *Camelus ferus* and *Camelus dromedarius* are similarly divergent.. Asparagine at position 365 is replaced by glycine in different species. Two more substitutions of leucine and histidine were observed at positions 4.1.93 and 400, respectively. The interactive behavior of both proteins (death domains) is significantly involved in the prevalence of AD^11^.

### Physiochemical analysis of DDs

A PepDraw online tool (http://www.tulane.edu/~biochem/WW/PepDraw/) was employed to check the physiochemical properties of DDs structures. The computational results showed that both DDs possessed isoelectric points 9.40 and 10.34, respectively. A net positive charge has been observed on both DDs because the sum of positively charged amino acids was larger than the negatively charged residues. Moreover, the hydrophobicity can be defined as the free energy associated with transferring a peptide from an aqueous environment to a hydrophobic environment like octanol. Our *in-silico* results showed that DDs of MADD and TNFR1 possessed hydrophobicity values 66.45 and 51.80 kcal/mol, respectively. It has been reported that the hydrophobic effect is the important driving force for the folding of globular proteins^34^. Therefore, the observed hydrophobic values may play a significant role in the understanding of folding and binding interactions in DDs.

The theoretical *pI* value of proteins is calculated by the accumulation of average isotopic masses and pK values of linear amino acids, respectively. Prior research data showed that proteins are distributed across a wide range of *pI* values (4.31 to 11.78)^35^. The MADD and TNFR1 DDs possessed pI values 8.26 and 5.87, respectively, which are comparable with the standard values. These predicted *pI* results show the accuracy and reliability of DDs structures. The grand average of hydropathy (GRAVY) value is the sum of hydropathy values of all residues in protein^36^. The prior research reports justified that the GRAVY negative and positive values show hydrophilic and hydrophobic behavior of protein structures^35^. The negative GRAVY values (-0.709 and -0.375) of both proteins (MADD and TNFRA1) domains indicate ahydrophilic behavior. Moreover, the aliphatic index value also showed the stability and relative volume occupied by aliphatic side chain residues. The predicted physiochemical properties showed the reliability, efficacy and stability of the protein structures. The predicted values of both proteins (MADD and TNFR1) domains are quite justifiable as compared to standard values which showed the accuracy of both structures as mentioned in Table 1. Furthermore, the overall ERRAT quality scores for both proteins were determined by ERRAT tool.

**Table 1:** Analysis of MADD and TNFR1 structures.

Physiochemical properties	MADD protein (Death domain)	TNFRA1 (Death domain)
Isoelectric point (pI)	9.40	10.34
Net charge	+ 1	+ 4
Hydrophobicity (kcal/mol)	66.45	51.80
Theoretical pI	8.26	5.87
Instability index	44.40	70.99
Aliphatic index	100.00	104.25
GRAVY	− 0.709	− 0.375
ERRAT Quality Factor (%)	100	100

### Structural analysis of MADD and TNFR1 death domains

The retrieved TNFR1 and predicted death domain of MADD were structurally evaluated to check the residual architecture using VADAR 1.8. The TNFR1 death domain contains 78% of helices 21% of coils and 27% of turns, whereas MADD death domain consists of 84% of α-helices, 15% of coils and 15% of turns, respectively. Furthermore, the Ramachandran graphs and values also confirm the reliability and efficacy of both death domains. The Ramachandran plots indicate that 98.99% of all residues of death domains in TNFR1 and 94.64% in MADD were present in favored regions, respectively (Supplementary data, Figs. S1).

## Molecular docking

### ZDOCK analysis for death domains of MADD and TNFRA1

To explore the core residues involved in the binding interaction of DDs, the docked complexes were analyzed using scoring values and residual binding interactions (hydrgen/hydrophobic). ZDOCK results are based on particular scoring functions^37^. Top ten ZDOCK predictions of docked complexes were selected and ranked based on scoring values mentioned in Table 2. The generated docked complexes showed different scoring values. and complex 1 (1052.41) was the most significant as compared to the rest of all docked complexes. It was also observed that complex 1 showed the best conformational position and good interactions between residues in both MADD and TNFR1.

**Table 2:** Docking energy values of docked complexes using ZDOCK.

Complexes	Scores	Euler angles			Grid positions		
1	1052.41	− 0.523599	1.732874	− 0.951685	12	95	97
2	1038.01	− 0.785398	1.194549	− 0.801900	10	92	98
3	1003.02	1.832596	1.247695	1.809890	10	93	92
4	1001.75	− 1.308997	1.510910	− 0.740472	6	87	97
5	998.758	− 1.047198	1.510910	− 0.740472	9	90	98
6	992.871	− 0.785398	1.510910	− 0.740472	11	92	98
7	985.988	− 0.785398	1.732874	− 0.951685	11	99	94
8	973.144	− 1.047198	1.982574	− 1.115518	11	97	94
9	971.887	− 1.047198	1.732874	− 0.951685	11	97	94
10	967.575	− 0.785398	2.138223	1.678323	12	99	98

*Euler angles (in radians) for rotating the (death domain-MADD) while grid positions describing the translation of the death domain-MADD concerning its starting point.

### ClusPro 2.0 docking analysis in MADD-TNFRA1 docked complexes

The best DDs docked complexes having good cluster size were analyzed to check the binding affinities of DDs. The predicted docked complexes having favorable surface complementarities are retained and scrutinized based on good docking energy values (kcal/mol). The cluster centers are ranked according to cluster sizes and best complex showed cluster size 142 having center and lowest energy score values 624.0 and − 711.8, respectively (Table 3).

**Table 3:** Docking energy values generated by ClusPro server.

Cluster	Members	Representative	Weighted score
0	142	Center	− 624.0
		Lowest Energy	− 711.8
1	56	Center	− 611.6
		Lowest Energy	− 711.5
2	55	Center	− 647.2
		Lowest Energy	− 674.7
3	52	Center	− 633.9
		Lowest Energy	− 673.5
4	47	Center	− 663.0
		Lowest Energy	− 664.2
5	45	Center	− 606.3
		Lowest Energy	− 663.5
6	45	Center	− 691.9
		Lowest Energy	− 691.9
7	44	Center	− 608.0
		Lowest Energy	− 696.0
8	41	Center	− 597.2
		Lowest Energy	− 693.0
9	35	Center	− 607.5
		Lowest Energy	− 694.4

### HawkDock analysis

**Figure 3:**
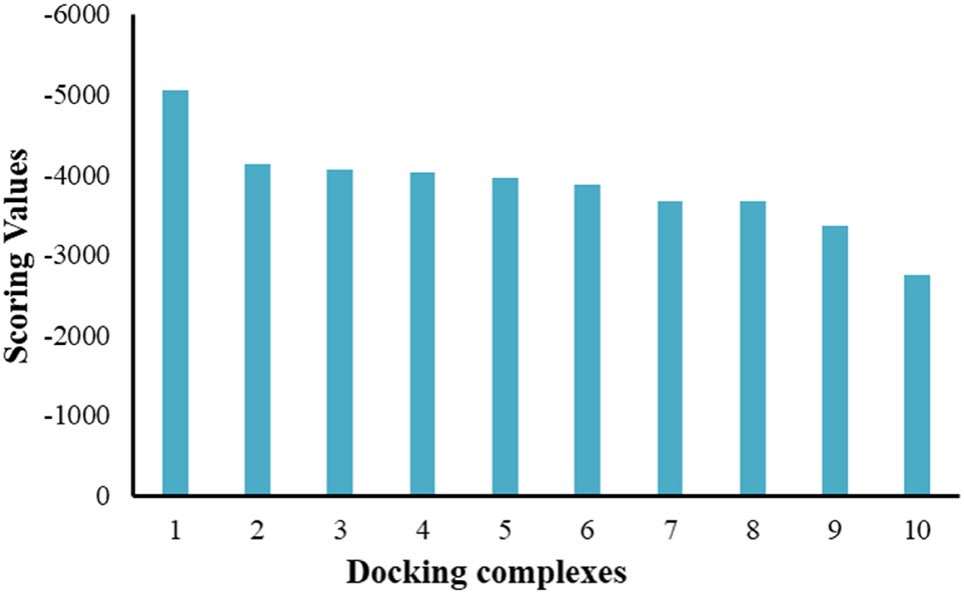
Scoring values and docking energy values (in kcal/mol) for 10 models generated by HawkDock.

To configure the computational binding efficiency of death domains, HawkDock docking server was employed and results were analyzed based on scoring values and interactive behavior. The generated docked complexes showed that complex 1 exhibited the highest scoring value (− 5000) as compared to other complexes. However, the rest of all remaining complexes 2–10 possessed − 4000 to − 3000 scoring values, respectively (Fig. 3).

### Binding pocket predictions of MADD and TNFR1 death domains

**Figure 4:**
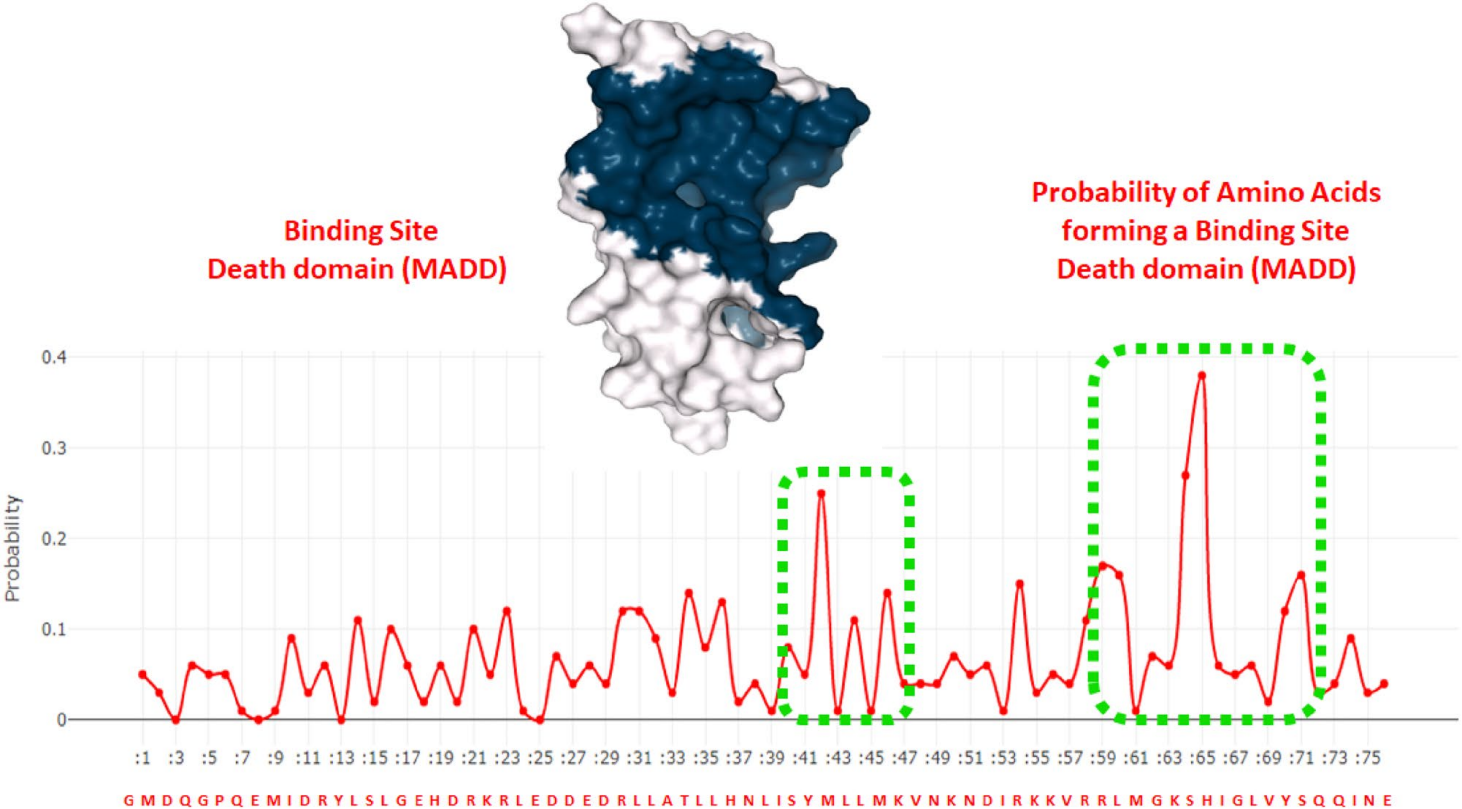
The binding pocket of MADD death domain showing probability of amino acids of forming a binding site.

Protein death domains are the structural and functional unit of macromolecules (protein/enzymes), which are involved in the activation of different signaling pathways^38^. To find out the binding pocket of the MADD death domain, an online server DEPTH was employed to compute the probablilty of forming binding sites by residues belonging to protein death domains (Fig. 4). Two major propensity peaks were observed for amino acid rang Leu39-Lys47 and Arg59-Gln73, respectively. The Tyr42 showed the highest binding site propensity 0.25 whereas surrounding residues Leu44 and Met46 exhibited probability values 0.11 and 0.14. Similarly, in other peaks, His65 showed the highest probability value 0.38. In this region different amino acids also depicted good probability values such as Ser64: 0.27, Arg50: 0.17, Leu60: 0.16, Tyr70: 0.12 and Ser71: 0.16, respectively.

**Figure 5:**
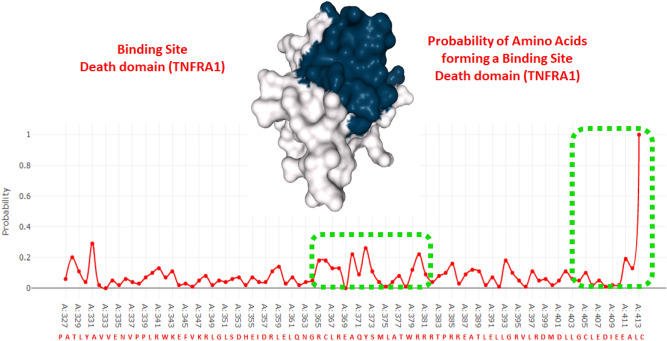
Binding pocket of TNFR1 death domain showing probability of amino acids of forming a binding site Binding interactions of MADD and TNFR1 death domains.

In TNFR1 death domain, two major probability peaks were observed for amino acid range Arg365-Thr382 and Leu405-Cys413, respectively. In Arg365-Thr382 range residues Ala370, Tyr372 and Arg380 exhibited probability values 0.22, 0.26 and 0.22. The other predicted binding pocket contains seven amino acids. In this region, Cys413 showed the highest probability value 1 compared to other amino acids. Couple of other residues Ala411 and Leu405 possessed probability values 0.19 and 0.10, respectively (Fig. 5).

**Figure 6:**
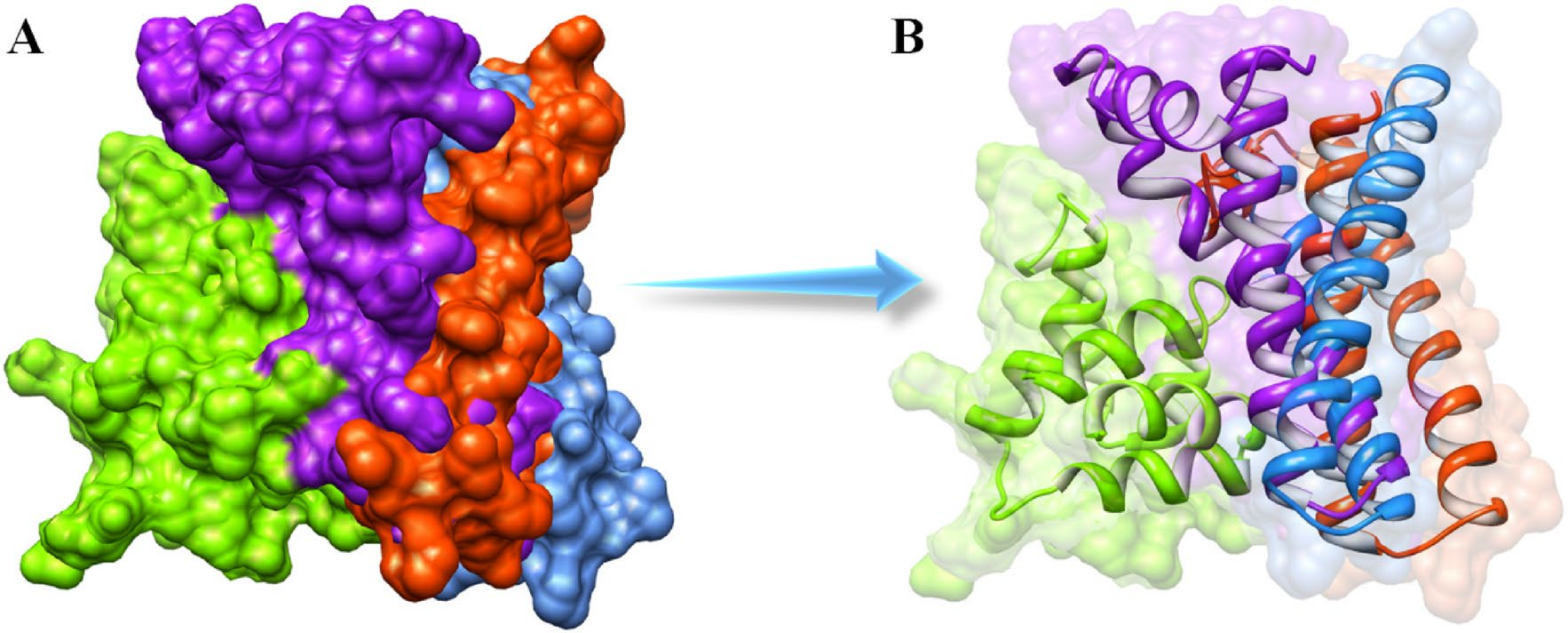
(**A**, **B**). The binding conformations of MADD and TNFR1 death domains. The TNFR1 death domain is represented in green color, whereas the MADD death domain of three docked complexes is highlighted in purple, blue and orange colors respectively.

The understanding of binding interactions between the death domains of MADD and TNFR1 is a significant approach for understanding the interactive behavior of proteins and their mediated downstream signaling pathways. Three best docked complexes were utilized to observe the unique pattern of the binding interactions between the MADD and TNFR1 death domains. The MADD death domain is composed of two helices joined together through a small loop structure and open at a distant end. This symmetry exposed a broader area and more chances of interactions with the target protein. In the ClusPro predicted (green-purple) complex, the TNFR1 death domain interacts with the central part of the MADD death domain. However, in HawkDock predicted (green–blue) complex both death domains showed different conformational positions in comparison to the ClusPro docking. The carboxyl terminal helix showed small deviation and N-terminal depicted a closer interactive behavior. A similar, binding conformational pattern was observed in the ZDOCK predicted struture compared with other docked complexes (green-orange). The interconnected loop regions of all three death domains were observed at a similar configuration where parts of opening helices showed different conformational patterns (see Fig. 6A and B).

### Binding analysis of death domains in docked complexes

The appropriate hydrogen/hydrophobic interactions among proteins are involved in multiple biological processes and other underlying molecular mechanisms in different diseases^39,40^. Furthermore, prior research data also showed that the stability of protein complexes depends upon the hydrogen bonds^41^. Moreover, in protein–protein docking analysis, hydrogen bonds play an important role in judging the best conformation position in the active region of a target protein. The most suitable hydrogen bond distance ranges from 1.9–2.5 Å; however, another study depicted this bond distance from 1.5 to 3.8 Å^42,43^. It has been observed that all the protein–protein docked complexes are unique in binding pattern and conformations.

In ClusPro predicted complexes, three hydrogen bonds were observed in DDs of the docked complex. The TNFR1 death domain residues positioned at Arg368, Glu410, and Asp400 were actively participating in hydrogen bond formation with MADD death domain residues Asp26, Se41 and Ag58, respectively. The Arg368 forms a hydrogen bond with Asp26 having a bond length of 2.61 Å. The acidic residues Asp400 and Glu410 of TNFR1 death domain directly formed hydrogen bonds with Ser41 and Arg41 having bond distances 1.97 Å and 1.79 Å, respectively. However, there are some different residues Glu406, Arg341, Glu409, asp407, Asn336, Pro339, Cys404, Leu401, and Glu335 of TNFRA1 and Lys50, Arg54, Glu25, Asp29, and His37 of MADD death domain, which are involved in the docked complex and enhance the stability of protein–protein interaction.

**Figure 7:**
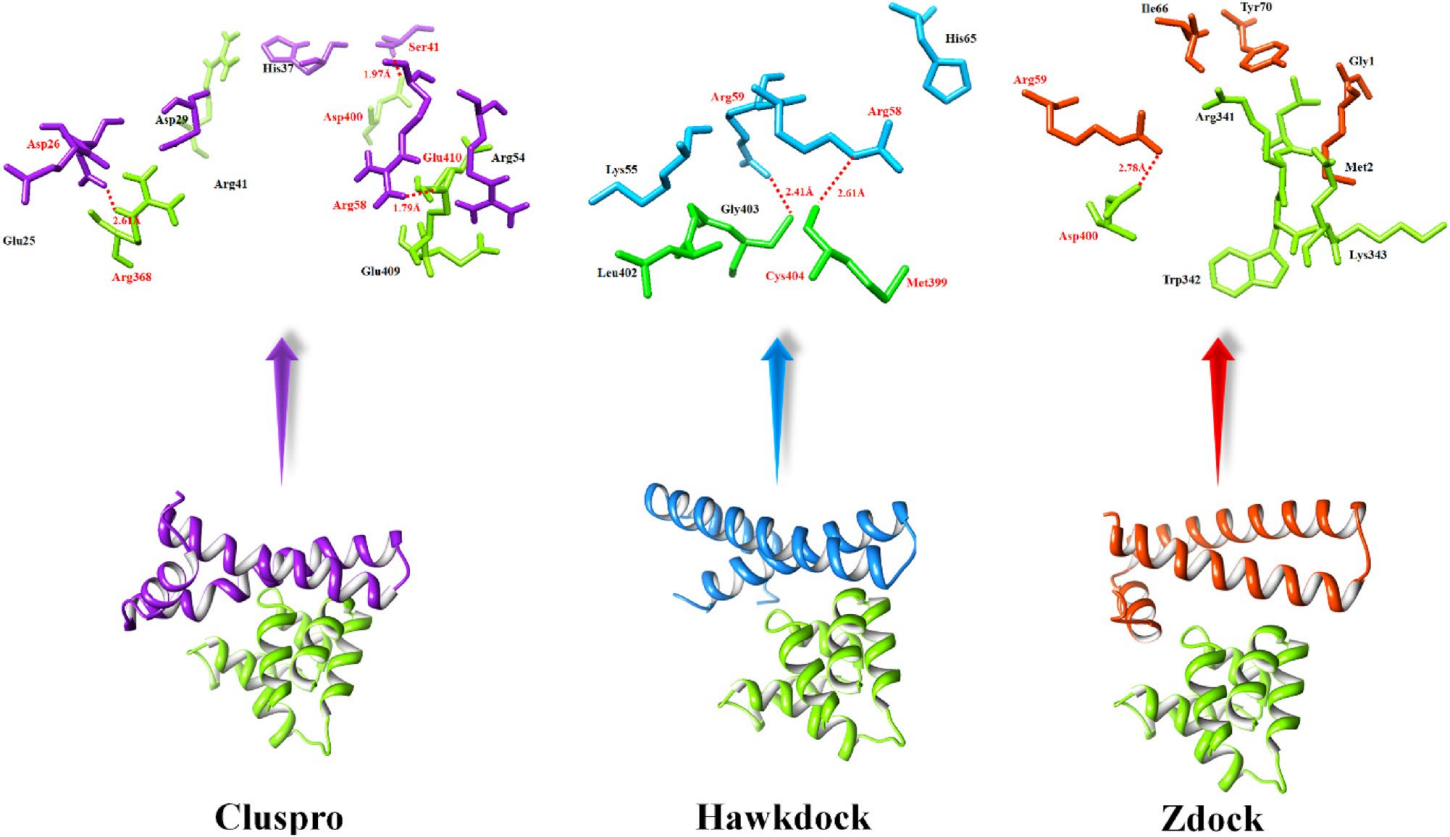
Docking interactions between DDs of TNFR1 and MADD. The surface structure of TNFRA1 is highlighted in green color whereas, MADD is justified in three different colors like purple, blue and orange-red, respectively. The red dotted lines represent hydrogen bond distances in angstrom (Å).

In the HawkDock predicted complexes, two hydrogen bonds were observed strengthening the docked complex at different residual positions. The TNFR1 death domain residues such as Met399 Leu402, Gly403, and Cys404 are actively participating in binding interactions with the MADD death domain residues Lys55, Arg58, Arg59 and His65, respectively. The binding pocket residues such as Arg58 and Arg59 form hydrogen bonds with Met399 and Cys404 with bond lengths 2.61 and 2.41 Å, respectively. In the binding pocket prediction results, Arg59 showed a good probability value and was also involved in hydrogen biding according to generated docking results (Fig. 7). Similarly, the ZDOCK-generated complexes of the DDs of TNFR1 and MADD were also analyzed based on conformation and residual interaction patterns. A single hydrogen bond was observed between Arg59 (MADD) and Asp400 (TNFRA1) with a bond length of 2.78 Å. Moreover, other residues such as Gly1, Met2, Ile66 and Tyr70 (MADD death domain) and Arg341, Trp342 and Lys343 (TNFRA1) were involved in hydrophobic interactions and strengthening the docked complex. The comparative analysis showed that the helical region of the MADD death domain has more potential to be actively involved in the activation of TNFR1 signaling pathway. The Arg58, Arg59 (MADD: death domain) and Asp400 (TNFR1) were the most common amino acid and key players involved in the stability of docked complexes and activation of a downstream signaling pathway (Fig. 7). Moreover, the common residues in all dockings are mentioned in Table 4.

**Table 4:** Residual analysis using hydrogen bonding.

Death Domains	Interactive residues of docked complexes		
	ClusPro	HawkDock	ZDOCK
TNFR1	Arg368, Asp400, Glu409, Glu410, Arg41,	Leu402, Cys404, Met399	Asp400, Trp342, Lys343, Arg341
MADD	Asp26, Asp29, His37, Ser41, Arg54, Arg58	Lys55, Arg58, Arg59, His65	Gly1, Met2, Arg59, Ile66, Tyr70

## MD simulation analysis of docked complexes

### Root mean square deviation and fluctuation analysis

**Figure 8:**
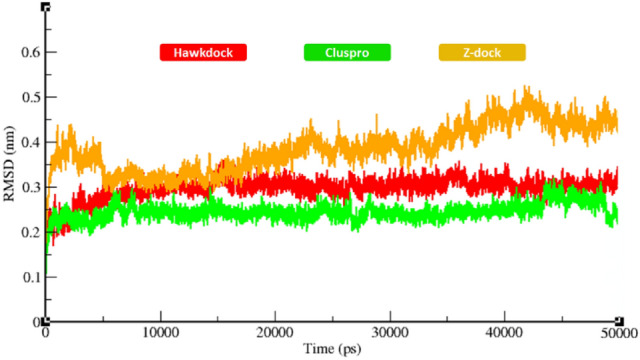
RMSD graph of all the docked structures during 50,000 ps simulation.

The assessment of protein backbone flexibility of best-docked complexes was carried out using MD simulation through RMSD/F, Rg and SASA graphs. The generated RMSD results showed that protein backbone deviations and fluctuations during 50 ns MD simulations. All the graph lines displayed slightly increasing trend with RMSD values ranging from 0.2 to 0.5 nm during 50 ns simulations. For predicted HawkDock models, the graph line (red) slightly fluctuated during simulations around RMSD value 0.3 nm. In the equilibration phase, RMSD increased from 0.2 to 0.3 nm at 5000 ps. However, after that time period a steady behavior was observed. This steady behavior represents a stable protein backbone in the docked complex. In the static phase of simulation, the graph line remained stable from 5,000 to 50,000 ps time period. In simulation of ClusPro predicted complexes, the same graph pattern as for HawkDock models was observed with exceptional deviations. Throughout the simulation, the RMSD value remained stable at 0.25 nm. At the simulation time point of 25,000 ps a small fluctuation was observed, however, the RMSD value ranged from 0.2 to 0.25 nm. Similarly, after that simulation time period graph line (green) showed stable behavior, except the terminal part of simulation at time at 45,000 ps; however, computed RMSD value is 0.25 nm. For models predicted by the third docking server (ZDOCK), the fluctuations pattern was evelaved with RMSD values ranging from 0.3 nm to 0.45 nm. In the equilibration phase (the first 5,000 ps), the graph line (orange) showed higher fluctuations within a range 0.3 nm to 0.4 nm. From time 5,000 to 15,000 ps RMSD was decreasing with time. After that, a small increasing trend was observed with RMSD continuously increasing with time. The comparative analyses showed that docked complexes remained stable with no large fluctuations for HawkDock and ClusPro predicted models. However, ZDOCK predicted complex remained slightly unstable during the simulation in comparison to HawkDock and CluPro predictedcomplexes (see Fig. 8).

**Figure 9:**
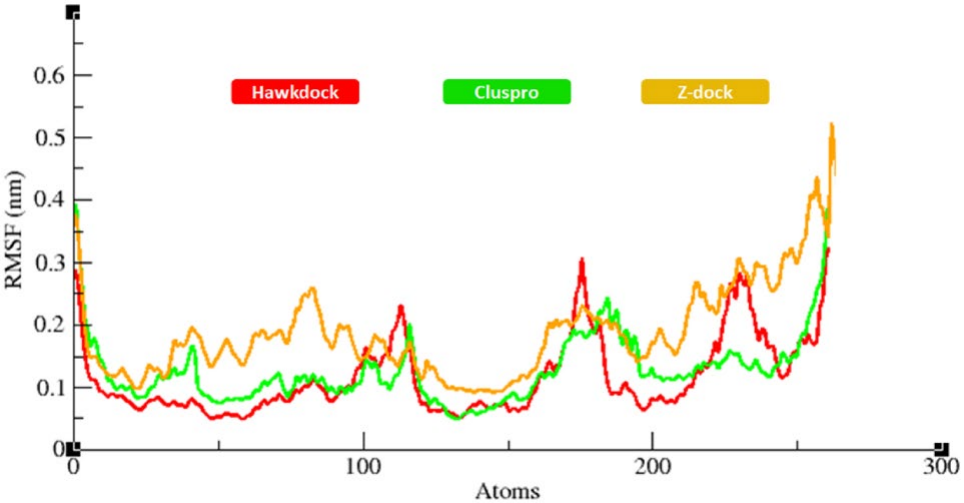
RMSF graphs showing fluctuations of protein backbone residues during 50,000 ps simulation.

The RMSF results of all docked complexes predicted by three different docking servers showed that protein structures dynamically fluctuate. Small fluctuation peaks with RMSF value 0.5 nm were observed in the loop regions of death domains of the MADD protein. The generated RMSF graph showed that the docked structure predicted by ZDOCK exhibit larger fluctuations than complexes predicted by two other servers. However, the rest of graph lines exhibited little variations in loop regions. The comparative results showed that both HawkDock and ClusPro-predicted complexes were more stable than the ZDOCK-predicted docked complex (Fig. 9).

### Radius of gyration and solvent accessible surface area analyses

**Figure 10:**
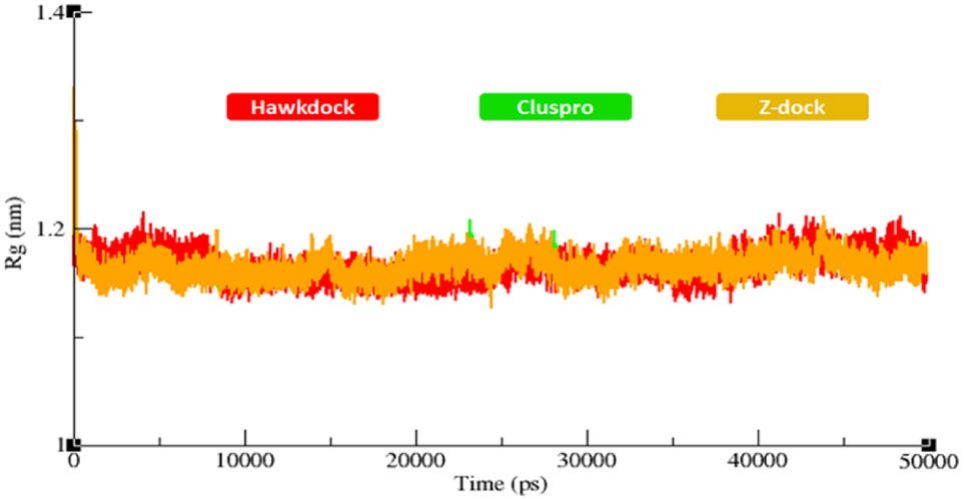
Rg of all the docked structures from three docking servers during 50000 ps simulation.

**Figure 11:**
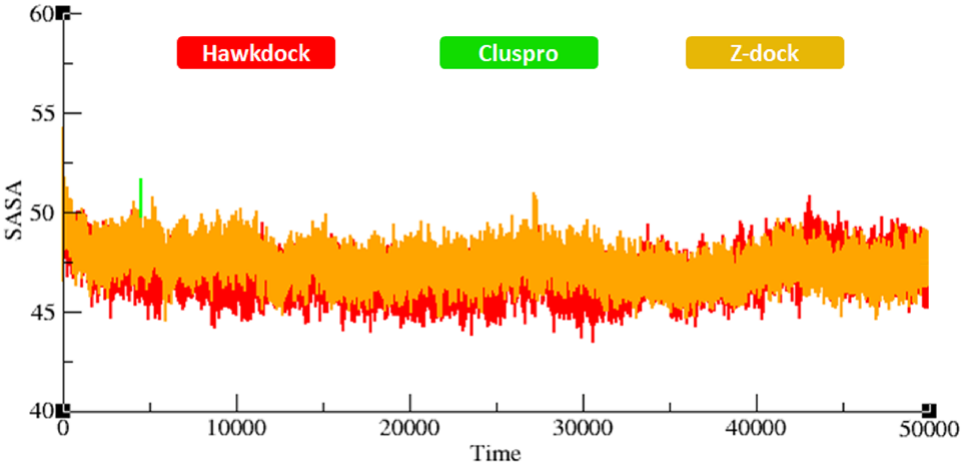
SASA of all the docked structures from three docking servers during 50000 ps simulation.

The compactness in the protein structures is calculated by the radius of gyration (Rg). It has been observed that perfectly folded structures show the relatively steady value of Rg, whereas the disordered regions of proteins show higher fluctuations of Rg in the MD simulations. The generated results depicted that Rg values of all the docked proteins showed little variations from 1.2–1.3 nm. The overall results showed that all graph lines were centered around 1.2 nm (Fig. 10). The solvent-accessible surface areas (SASA) were also observed and shown in Fig. 11. Results showed that the values of SASA of the docked complexes from the three different docking servers are centered around 50 nm^2^ in the MD simulations carried out for 50,000 ps.

## Mechanistic signaling pathways

### NF-kB mediated TNF signaling in AD

The activation of NF-kB in the brains of AD patients is associated with the activation of TNF-α in Aβ peptides and astrocytes implicated in neuroinflammation. TNF-α signaling can elicit various cellular responses through TNFR1 and TNFR2 depending on a variety of factors including the cellular metabolic state and adaptor proteins^44^. The differences in various TNFR1 associated signaling pathways such as NF-kB, p38, c-Jun N- terminal kinase (JNK) and ceramide/sphingomyelinase signaling pathways lead to multifarious processes, such as proliferation, inflammation, cellular migration, apoptosis and necrosis. TNFR2 mediated signaling also activates the inflammatory and apoptotic pathways through interaction with adaptor proteins of cIAPs and NF-kB pathway^45^.

**Figure 12:**
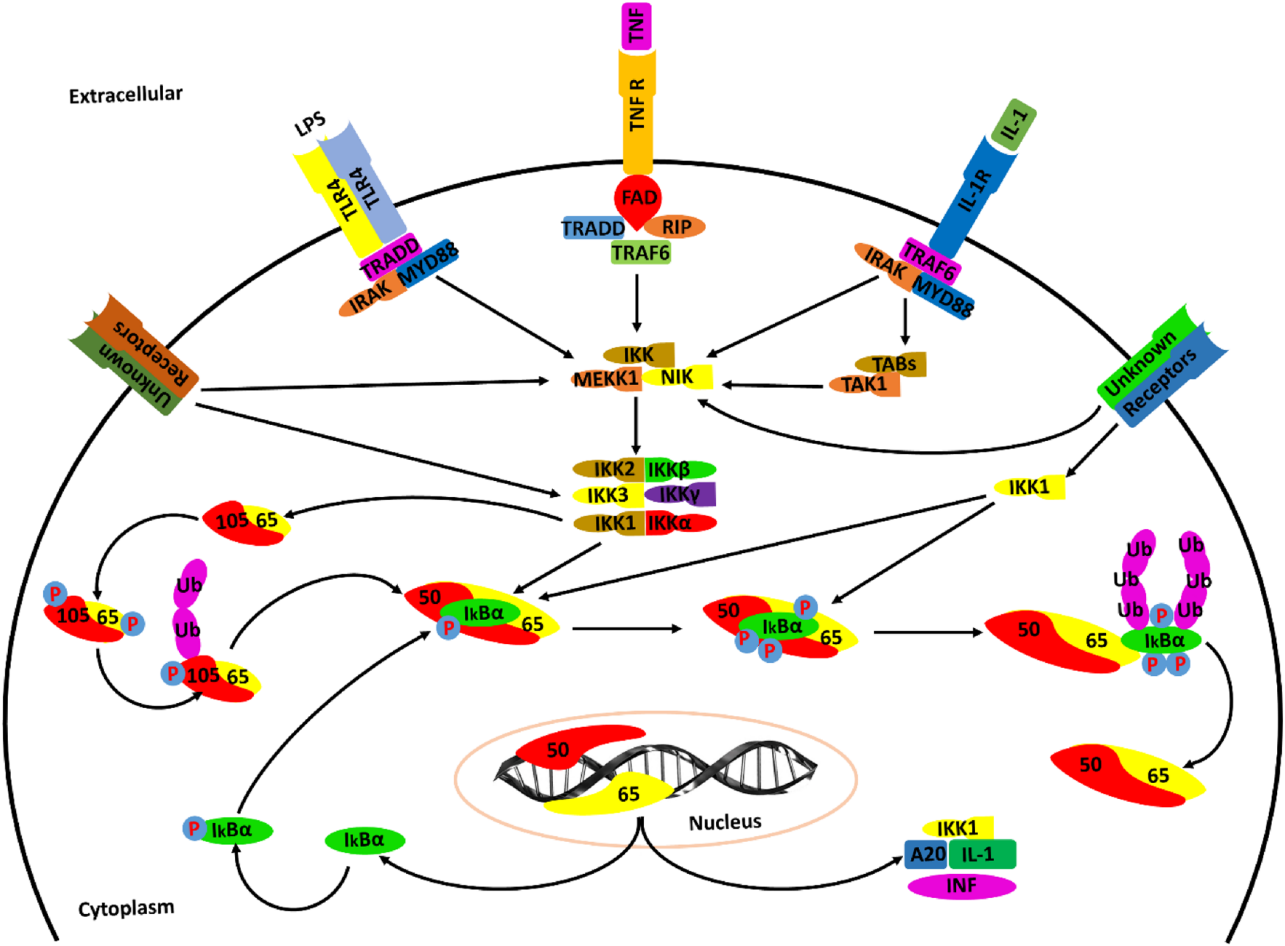
NFkB is the major cascade cross talked by TNF signaling. TNF mechanistic signaling occurs primarily via its TNFR1 and TNFR2 receptors ubiquitously expressed in brain tissues in AD patients. The extracellular domain of TNF receptors is immediately characterized by the presence of cysteine-rich CCG domains that mediate the specific ligand receptor interaction. Other transcription factors such as p38 and c-Jun terminal kinase (JNK) is also activated through downstream regulation of TRAF2 and RIPK1. TRADD uses its death domains to bind to the TNFR1 complex where it is most likely to express as a scaffold to recruit TRAF proteins and FADD. MAPK signaling is also mediated through downstream stimulation of TNFR1 and TRAF 3. Upon activation of a subset of neuronal inflammation superfamily, TRAF 3 is degraded. This event causes the activation of MAP3K14, interleukin-1 and Toll-like receptors pathway and subsequent activation of IKK alpha unit which upregulates the alternative NFkB signaling.

The neutrophin growth factor (NGF) promotes cell survival supported by increased immunoreactivity of p65 subunit in hippocampal cultured neurons, peripheral glial cells and pheochromocytomas. Numerous kinases associated with memory cell mechanisms such as protein kinase A (PKA), protein kinase C (PKC) and CaMKII are responsible for the activation of NF-kB. PKA phosphorylation of p65 subunit of NF-kB is directly associated with the activation of IkB which in turn interacts with p65 (Fig. 12). Therefore, PKA is a genetic target of NF-kB in neurons. CaMKII is abundantly present at synapses; however, CaMKII members including CaMKIV are predominantly located in nuclei^46^. Recent studies suggested that NF-kB implication in pertaining the homeostasis of neurons in the brain adapt their activity in efforts to preserve the stability of neuronal networks’ mechanisms. Therefore, the immunoreactivity of the p65 subunit is known to be elevated in several neuronal elements including neurofibrillary tangles and dystrophic neurites, particularly in AD patients. Although several studies present elevations in NF-kB complexes in AD brains, some studies also reported decreased expression of NF-kB associated p65 localization in both neurons and microglial cells around the mature cortical brain tissues in AD patients^47^.

Ceramide a lipopolysaccharide found in plasma membranes of neuronal cells also has the ability to stimulate NF-kB in Schwann cells similar to that observed in TNF-α signaling cascade. The hippocampal neurons were observed to express the upregulated expression of calcium sensing receptor CaSR activated by increased concentration of TNF-α after a 24-h exposure of ceramide. The combinatorial application of ceramide with TNFR1 demonstrates the activation of Ca^2+^ in Schwann cells. As a result, AD patients tend to exhibit higher rates of cognitive decline which is clearly pointing the role of NF-kB in cytokine boost and inflammation progression in AD^48^.

### Downregulation of DENN/MADD, a TNF receptor binding protein, correlates with neuronal cell death in Alzheimer brain

**Figure 13:**
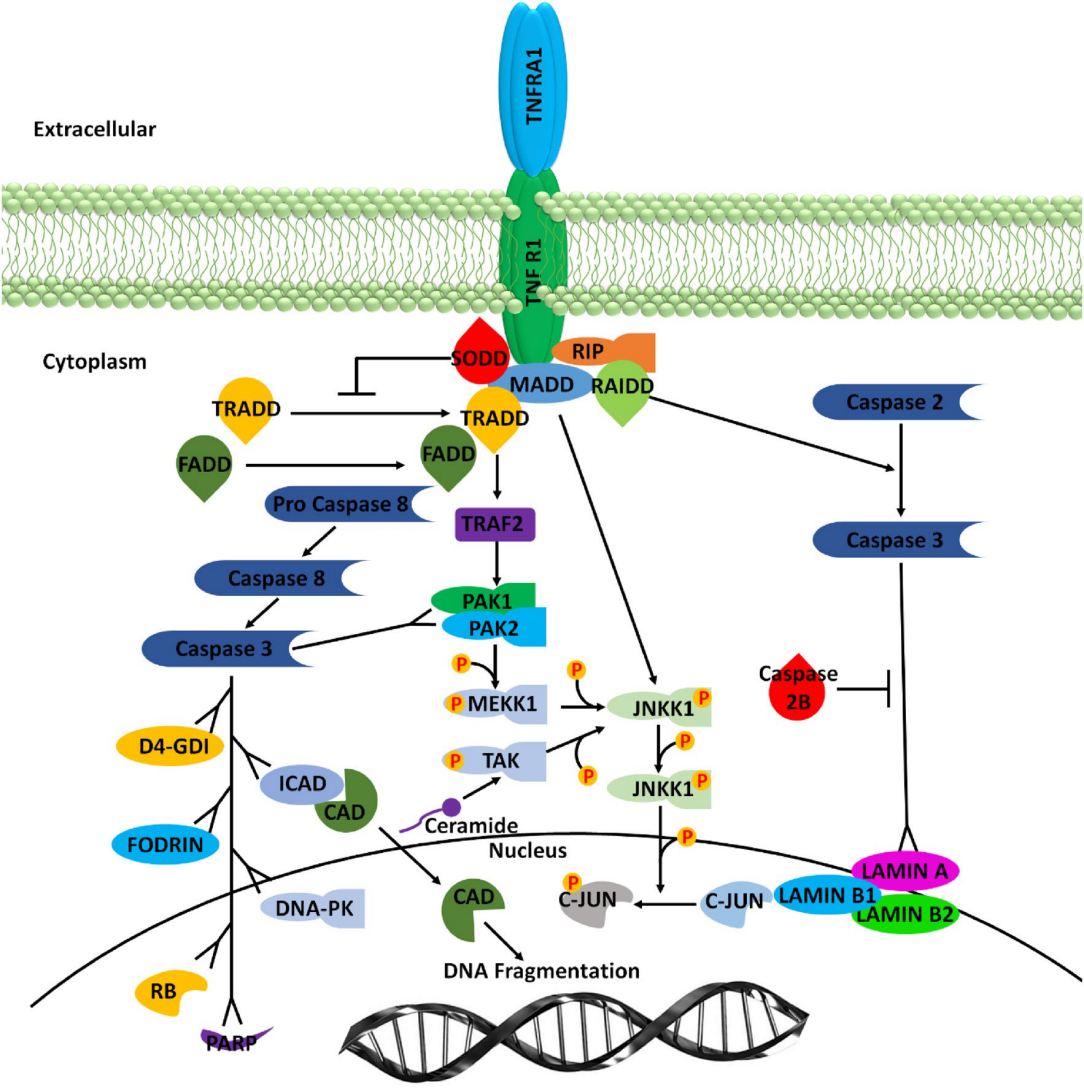
MADD is a key signaling protein complex localized in the cytosol which exerts its biochemical signaling via different binding factors. In humans MADD is the death domain protein that regulates the expression of Rab3 GDP/GTP exchange protein under normal circumstances via Ca^2+^ dependent neurotransmitter release from the synaptic cleft and exocytosis. MADD is also reported to block the apoptosis process in neuronal cells in cytotoxic conditions of AD.

DENN (differentially expressed protein in normal and neoplastic cells) domain is a poorly characterized protein module associated with MADD (MAP kinase-activating death domain protein) conserved throughout the apoptotic signaling pathway^49^. MADD propagates the apoptotic signal at a higher level in neoplastic cells as compared to normal cells transduced by the cytoplasmic adaptor proteins such as TNFR1. The altered expression of TNFR1 binding proteins in AD promotes JNK activation via apoptosis signaling kinase (ASK1) which is observed to be downregulated in AD^50^. The associated DENN overexpression in non-neuronal culture systems leads to the activation of both extracellular-regulated kinase (ERK) and JNK. Since MAPK are upregulated in AD. whereas MADD and TRAF 2 are inhibited, therefore these transcriptional factor proteins do not participate in JNK-ERK mediated cell death or survival cascades. Several studies suggested increased expression of TRADD and TNFR1 that results in optimal cell death in AD tissues^51^. These facts reveal that receptors such as TNFR1 are involved in promoting cell death mediated by MADD/DENN signaling in neuronal cells in brain tissues of AD patients. The Aβ plaques in AD show residual MADD/DENN expression in neurites which is then coupled with nuclear localization of overexpressed JNK (Fig. 13). AD transgenic mouse model Tg2576 also expressed decreased levels of MADD/DENN in cortical brain regions of AD inflammation^52^.

MADD also works in association with Ca^2+^ mediated exocytosis as well as neurotransmitter release^53^. Therefore, in humans, MADD plays a potent physiologic role as GEP (Guanine nucleotide exchange protein) in neuronal cells of synaptic vesicles. The DENN/MADD, therefore, is observed to post dock synaptic exocytosis leading to the decreased release of neurotransmitters including acetylcholine in AD brain tissues which is a potent feature of AD^54^.

## Conclusions and discussion

The discovery of the apparent dual role of TNF through its two receptors has initiated extensive research into new possibilities to treat neuroinflammation, which is a common hallmark of neurodegenerative diseases. Recent studies have explored the binding behavior of MADD against TNFR1 particularly through death domains as a possible cause of AD^55^. Our molecular docking results also justified that there is a close interaction between DDs of TNFR1 and MADD and common interactive residues Asp400 and Arg58 and Arg59 were observed in structures predicted by all three docking servers. Aspartic acid is a negatively charged residue involved in the biosynthesis of proteins^56^. However, arginine is a positively charged residue having the tendency to interact with oppositely charged amino acids^56^. These oppositely charged residues (positive–negative) have the potential to form an interaction that may stabilize both DDs in the docked complexes. The amino acids Asp400 (TNFR1), Arg58 and Arg59 (MADD) may be considered as key players in the activation of downstream signaling pathways, and can be used as new targets to design novel pharmaceutical agents against AD. Therefore, the development of specific TNFR1 antagonists and solTNF inhibitors (ATROSAB and XPro-1595) that ameliorate inflammation and apoptosis, and TNFR2 agonists that enhance neuro-regeneration and tissue homeostasis, are promising strategies to treat neurodegeneration.

Protein interactions initiate the downstream signaling pathways and are involved in the occurance of neurological diseases^57,58^. The decreased flexibility of protein backbone is considered a major hallmark for checking the stability of docked complexes using MD simulation^56^. The protein–protein interactions of biomolecules mediate some conformational changes in the proteins’ structure which lead to activation of mediator proteins in signaling pathways. Therefore, MD simulation generated RMSD/F, Rg and SASA graphs showed good stability of the TNFR1-MADD complexes. It has been observed that the standard value for RMSD for any particular protein ranges from 0–0.5 nm ^59,60^. In comparison with this standard value, RMSF graphs of all three dockings showed comparable results which depicted the stability of docked TNFR1-MADD (DDs) complexes. Furthermore, the Rg and SASA (Å^2^) results also showed the compactness of protein structure and conformational dynamics. It has been observed that the Rg graphs and values remained the same and little fluctuations have been reported in Figs. 10 and 11, respectively. The interactive residues showed little variations in the graphs and were supposed to be key factors that cause conformational dynamic variations. However, the overall effect has remained stable and the selected conformation could play an important role for understanding the AD mediated signaling cascade of TNFR1-MADD.

In future research, the identification of common amino acids would help identify the death domain interactive and conformational behavior and its involvement in the activation of TNFR1 and MADD signaling cascade in AD. A better understanding of DDs conformational behavior may help identify the particular target through which this signaling pathway can be deactivated to cure AD patients. Moreover, such death domains could also be used as a novel target for the development of new therapeutical agents for the treatment of AD. However, high speed processing systems are required to design all these signaling pathways modelings.

## Supplementary Information

Supplementary Information.
