# Supplementary material for: Mechanistic insights into TNFR1/MADD death domains in Alzheimer’s disease through conformational molecular dynamic analysis
Source: Sci Rep. 2021 Jun 10;11:12256. doi: 10.1038/s41598-021-91606-4 (PMC8192743; doi:10.1038/s41598-021-91606-4)
Supplement: Supplementary file 1 — Supplementary Information. [file 41598_2021_91606_MOESM1_ESM.pdf]

Supplementary data

Mechanistic insights into TNFR1/MADD death domains in Alzheimer's disease Through Conformational Molecular Dynamic Analysis

Mubashir Hassan[1*], Sara Zahid[1], Hany Alashwal[*2], Andrzej Kloczkowski[3,4] Ahmed A. Moustafa[5,6],

[1]Institute of Molecular Biology and Biotechnology, The University of Lahore, Pakistan.

[2]College of Information Technology, United Arab Emirates University, Al-Ain, 15551, UAE.

[3]Nationwide Children's Hospital, Battelle Center for Mathematical Medicine, Columbus, OH 43205 USA.

[4]Department of Pediatrics, The Ohio State University College of Medicine, Columbus, OH 43205, USA.

[5]Department of Human Anatomy and Physiology, the Faculty of Health Sciences, University of Johannesburg, South Africa

[6]MARCS Institute for Brain and Behaviour and [2] School of psychology, Western Sydney University, Sydney, New South Wales, Australia.

Corresponding Address

Dr. Mubashir Hassan

[1]Institute of Molecular Biology and Biotechnology, The University of Lahore, Pakistan

Email: mubashirhassan_gcul@yahoo.com

Dr. Hany Alashwal

Dr. Hany Al Ashwal, College of Information Technology, United Arab Emirates University, Al-Ain 15551, United Arab Emirates
Email: halashwal@uaeu.ac.ae

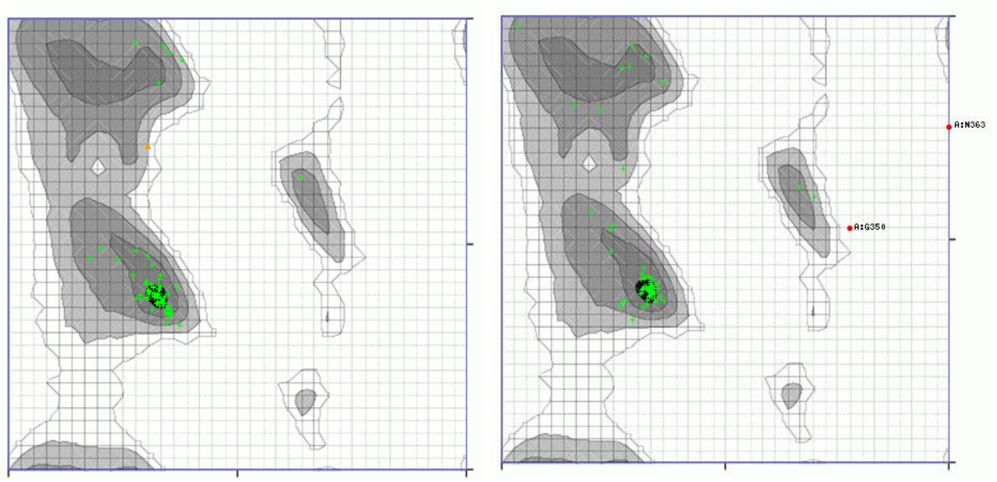

Figure S1. Ramachandran graphs of MADD and TNFRSF1A death domains
